# Design framework for polarization-insensitive multifunctional achromatic metalenses

**DOI:** 10.1515/nanoph-2021-0638

**Published:** 2022-01-04

**Authors:** Jacob T. Heiden, Min Seok Jang

**Affiliations:** School of Electrical Engineering, Korea Advanced Institute of Science and Technology, Daejeon 34141, Republic of Korea

**Keywords:** achromatic focusing, focused vortex-beam, metalenses, metasurface

## Abstract

Controlling the wavefront of light, especially on a subwavelength scale, is pivotal in modern optics. Metasurfaces present a unique platform for realizing flat lenses, called metalenses, with thicknesses on the order of the wavelength. Despite substantial effort, however, suppressing the chromatic aberrations over large operational bandwidths of metalenses still remains a challenge. Here, we develop a systematic design method enabling a simultaneous, polarization-insensitive control of the phase and the group delay of a light beam based on libraries of transmission-mode dielectric meta-elements. Mid-infrared achromatic metalenses are designed and theoretically analyzed to have diffraction-limited focal spots with vanishing chromatic aberrations in the operating wavelength range of 6–8.5 μm, while maintaining high focusing efficiencies of 41% on average. The proposed methodology, which can be used as a general design rule for all spectra, also provides a versatile design scheme for ultrashort pulse focusing and achromatic vortex-beam generation (orbital angular momentum), representing a major advance toward practical implementations of functional metalenses.

## 1 Introduction

Controlling light propagation through media is essential in light-energy-delivery and imaging systems. Conventional refractive optical components, enabling control of the optical wavefront, typically rely on a gradual phase accumulation during light propagation in a bulk material [1]. Consequently, the devices are inherently voluminous and laborious to manufacture, which severely limits their usage in densely integrating photonic systems to the nanoscale.

Metasurfaces composed of subwavelength-spaced structures at a planar surface provide unprecedented control over the properties of the electromagnetic field, including amplitude, phase, and polarization, and are thus attracting increasing attention [2], [3], [4]. This unique approach, for tailoring the optical response, has been utilized for numerous applications in the infrared and visible spectrums, including holograms [5], [6], [7], [8], [9], polarimeters [10], [11], [12], polarization elements [13], [14], [15], [16], and flat lenses (metalenses) [9, 17], [18], [19], [20], [21], [22]. Metalenses have especially attracted significant interest due to their applicability in compact imaging systems for both consumer and industry products encompassing cameras, microscopy, and lithography. However, such metalenses will generally suffer from considerable chromatic aberration, which can be attributed to two separate factors: resonant or guided light confinement, and periodic lattice dispersion. Substantial effort has been dedicated to combating this issue and developing achromatic focusing metalenses in the visible [23], [24], [25], [26], [27], [28], [29], near-infrared [30], [31], [32], [33], [34], [35], and mid-infrared (MIR) spectrums [36, 37]. Despite this effort and several works having been dedicated to analyzing the fundamental limits of achromatic metalenses [38, 39], an explicit design framework is still missing.

In this work, we analytically describe how the spectral degree of freedom C(ω) [33, 34, 36, 40], which modulates the reference phase at each design frequency, can be optimally tailored to fit a library of nanostructures having fixed group delays and group delay dispersions. The proposed methodology – which can be used as a general design rule for all spectra – further clarifies the phase range (hence the fundamental limits) that the metalens can cover while maintaining chromatic aberration correction. We verify our framework by generating a library of hexagonal silicon meta-elements and employing the library and methodology to design a set of metalenses. These metalenses exhibit diffraction-limited, polarization-independent focusing in transmission mode across a 2.5 μm wide bandwidth in the mid-infrared range (6–8.5 μm) with an average focusing efficiency of 41% over the entire bandwidth and a maximum efficiency of 54%. The performances of the metalenses are competitive with the current state-of-the-art designs (see Supplementary information section S1 for comparison).

Moreover, we demonstrate that the approach facilitates the design of achromatic metalenses capable of focusing ultrashort femtosecond pulses and generate focused vortex-beams carrying a specific orbital angular momentum (OAM). Broadband achromatic metadevices such as the ones we present can be employed in diverse practical applications including optical communications [41], endoscopy [42], depth-sensing [27, 28], and virtual and augmented reality [43]. In particular, in the MIR, achromatic metalenses could be well suited for integration with other nano-optical components for molecular sensing or bioimaging [35, 44].

## 2 Results and discussion

**Figure 1:**
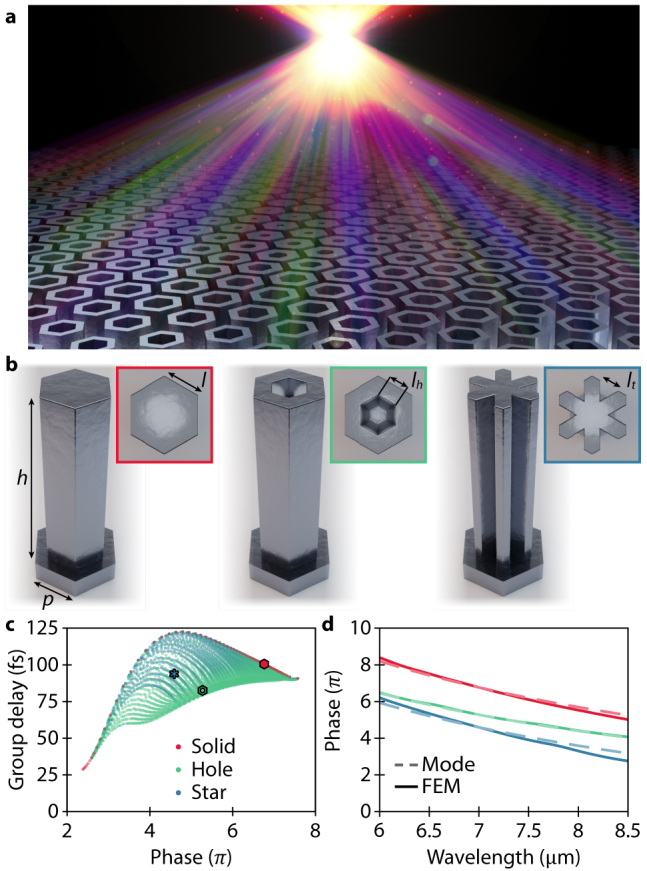
Principle of designing an achromatic metalens. (a) An achromatic metalens with dispersionless focusing. (b) Schematic of three different metalens elements consisting of Si nanopillars with the height of *h* = 8 μm and unit cell periodicity *p* = 2 μm. The displayed shape of elements has varying in-plane geometrical parameters (*l*, *l*_*h*_, *l*_*t*_). (c) Phase and group delay for all elements in our library at the center frequency *ω*_0_ = 42.62 THz (*λ* ≈ 7 μm). (d) A comparison of phase response for different meta-elements from eigenmode solutions versus finite-element method (FEM) calculations. The red curve represents a “solid” element; the green a “hole” element; and the blue a “star” element whose library positions are specified in (c).

The principle of designing an achromatic metalens revolves around accurate phase control at any position on the lens and is summarized in Figure 1. The required relative phase, provided by each metalens element, to achromatically focus a broadband incident beam in a diffraction-limited spot is given by the generalized Snell’s law, and must follow the relation:

(1) $$\varphi \left(r,\omega \right)=-\frac{\omega }{c}\left(\sqrt{r^{2}+f^{2}}\right)+C\left(\omega \right)\text{,}$$

where ω , c , r , and f are the angular frequency, speed of light, radial coordinate, and focal length, respectively. This evidently frequency-dependent phase profile indicates that a certain phase $\varphi \left(r,\omega_{0}\right)$ , group delay $\partial \varphi \left(r,\omega_{0}\right)/\partial \omega$ , and higher-order dispersion terms $\partial^{x}\varphi \left(r,\omega_{0}\right)/\partial \omega^{x}$ all need to be satisfied at every coordinate of the lens in order to remove chromatic effects (Figure 1(a)). We note that the group delay can be interpreted as the time delay that a wave packet experiences when it passes through a certain position of the metalens. To make wave packets from different positions arrive at the focal point simultaneously, the center of the metalens must exhibit higher group delay compared to the peripheral to compensate for the associated path length difference.

In previous studies of achromatic metalenses, the phase term has usually been decoupled from the higher-order terms by using the principle of the Pancharatnam–Berry phase [24, 25, 36, 37]. However, as also discussed in Ref. [28, 33], the designed lenses are inherently polarization-dependent, i.e., the incident light is required to have a specific circular polarization in order for the lens to focus. This polarization-sensitive limitation can be overcome by using symmetric pillars – here we utilize structures based on a hexagonal shape to provide the most unimpaired filling of the grid and minimize meta-element variance across the metalenses.

As illustrated in Figure 1(b), three different silicon pillar types, with height *h* = 8 μm and unit cell periodicity *p* = 2 μm, but varying in-plane geometrical parameters ( l , $l_{h}$ , $l_{t}$ ), are considered. Here *l* describes the side-length of each individual meta-element; *l*_*h*_ is the inner side-length of the “hole” elements describing the hole size; and *l*_*t*_ is the indent of an equilateral triangle on each side of the “star” element. Each pillar is modeled as a truncated dielectric waveguide with a frequency-dependent effective refractive index $n_{\text{eff}}\left(\omega \right)$ . Following this method, and neglecting reflection, the phase delay of the transmitted light that will be accumulated as it propagates through the structure is $\varphi =\left(\omega /c\right)n_{\text{eff}}h$ . Since $n_{\text{eff}}$ of the fundamental guided mode only weakly depends on ω , φ(ω) can be linearly interpolated with an average root-mean-square error of 1%. In this manner, a library containing 4040 meta-elements, each with a respective phase and group delay, is generated (Figure 1(c)). The mode calculation of each element took approximately 10 s, and thus the full library was generated in about 11 h. The waveguide model has an excellent agreement with full-wave simulations, as illustrated in Figure 1(d), which shows a comparison of the phase, induced by a meta-element, using the eigenmode solver and finite-element method (FEM, COMSOL Multiphysics 5.4). Furthermore, φ(ω) – obtained through the waveguide model – can be linearly interpolated with an average root-mean-square error of 1% effectively rendering the group delay dispersion to be zero (see Supplementary information section S2).

**Figure 2:**
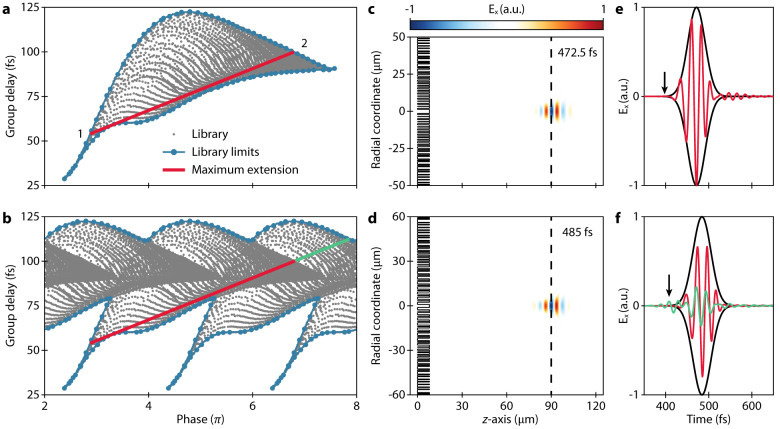
Framework for designing an achromatic metalens. (a) Phase and group delay for all elements in the presented library, distinctive by the grey dots, with the blue lines defining the upper and lower boundary of the library and the red line displaying the maximal phase range that the library can cover. (b) The same library is duplicated every 2*π* enabling a longer phase range, i.e., red line plus the additional green line. (c, d) Simulated normalized electric field (*x*-component) of a 25 fs optical pulse as it is focused at the focal point (*f* = 90 μm) with the a-type metalens design (NA = 0.49) and b-type metalens design (NA = 0.55), respectively. (e, f) Simulated normalized electric field (*x*-component) at the focal point as a function of time for the a- and b-type metalens design, respectively. The pulse in (f) is split into the component attributions of the red and green line from (b).

Figure 2(a) shows the meta-element library again (grey dots). Evidently, the library can be described by an upper and lower boundary, modeled as a polynomial interpolation (blue lines). To satisfy Eq. (1), the group delay $\partial \varphi /{\partial \omega |}_{\omega_{0}}$ should be directly proportional to the phase $\varphi (\omega_{0}$ ) with a $1/\omega_{0}$ slope, which can be represented as a straight line in Figure 2(a). The optimal phase range, which can be covered by the library, can be readily found by choosing the *y*-intercept of the straight line such that the length of the line segment enclosed in the library boundary gets maximized. The red line in Figure 2(a) shows the resulting optimal phase coverage for our hexagonal meta-element library, $\Delta \varphi =\varphi_{2}-\varphi_{1}$ , where the subscripts 1 and 2 denote the intersections between the line and the library boundaries. This phase range line clarifies the exact C(ω) required to achieve achromatic focusing and imaging, and thus acts as an analytical foundation for this specific engineering problem:

(2) $$C\left(\omega \right)=\frac{\omega }{c}f+\varphi_{2}\left(\omega_{0}\right)+\frac{\partial \varphi_{2}\left(\omega_{0}\right)}{\partial \omega }\left(\omega -\omega_{0}\right)\text{.}$$

We note that the scheme can be used as a general design framework for achromatic metalenses operating at various spectra. Advanced from the previous analytical approach suggested by Shrestha et al. [33], which addressed the problem of negative group delay by engineering C(ω) , our method offers a more thorough and complete way to optimally utilize a given meta-element library. Moreover, the proposed scheme is much more convenient and efficient compared to the previous approaches based on computationally-heavy numerical optimization algorithms such as particle swarm optimization [23].

It is noteworthy that the library is repeatable for every 2*π*, further extending the phase range that the library can cover, warranting a larger numerical aperture (NA, green line in Figure 2(b)). This is an approach utilized in essentially any metalens design. However, it has not been fully investigated how such a library repetition affects the performance of the metasurface for a pulsed input. To analyze this, we compare two metalenses: one designed from the original library shown in Figure 2(a) (called a-type) and the other utilizing the extended library allowing 2*π* repetition shown in Figure 2(b) (called b-type). The two metalenses are designed to have the same focal length *f* = 90 μm and library-limited diameters of *D* = 100 μm (NA = 0.49) and *D* = 120 μm (NA = 0.55) for the a- and b-type, respectively. For both cases, only “hole” elements are employed, and one “solid” element is used for the center of the lens. This is sensible because the predominant elements in the low group delay region of the library are “hole” elements and a drastic change in element geometry is more likely to cause unexpected inter-element coupling. The exact fitting procedure employed is further described in Supplementary information section S3.

The time-dependent propagation of a 25 fs optical pulse impinging on the a- and b-type lenses are simulated using the three-dimensional finite-difference time-domain (FDTD) method. Figure 2(c) and (d) shows the electric field snapshots of the a- and b-type lens systems taken at *t* = 472.5 and 485 fs, respectively. Note that it takes a longer time for the b-type lens to focus the pulse because of its larger diameter. Interestingly, the snapshots reveal a slight discrepancy in the wavefront propagation caused by the two different lenses. The b-type lens results in a more elongated pulse compared to the a-type lens at the focal point, but the observable aberration is modest. We attribute this to two reasons: first, the abrupt phase jump in the b-type lens system causes irregularities in the wavefront (Figure S5); second, the meta-elements from the extended part of the library exhibit non-ideal wave transmission behavior due to the excitation of higher-order modes and the non-linear dispersion terms as shown in Figure S6 (see Supplementary information section S4). The latter point becomes more evident by observing the electric field at the focal point in time as shown in Figure 2(e) and (f). The field of the b-type metalens is split into the contributions from the red and green line parts of the lens. The contribution from the red line, which is using the same part of the library as the a-type lens, displays a clear focusing behavior similar to the a-type lens. However, the contribution from the duplicated part of the library (the green line) arrives at the focal point earlier as indicated by the black arrow in Figure 2(f), causing a peculiar distortion of the waveform in the time-domain. The distortion of the waveform further increases as the metalens incorporates more meta-elements from the duplicated part of the library (i.e., increasing the length of the green design line) as shown in Figure S7. We anticipate that the problem of wave packet distortion of achromatic metalenses for pulsed inputs will become an important issue in ultrafast optical systems as the pulse duration reduces to a few optical cycles [45, 46].

**Figure 3:**
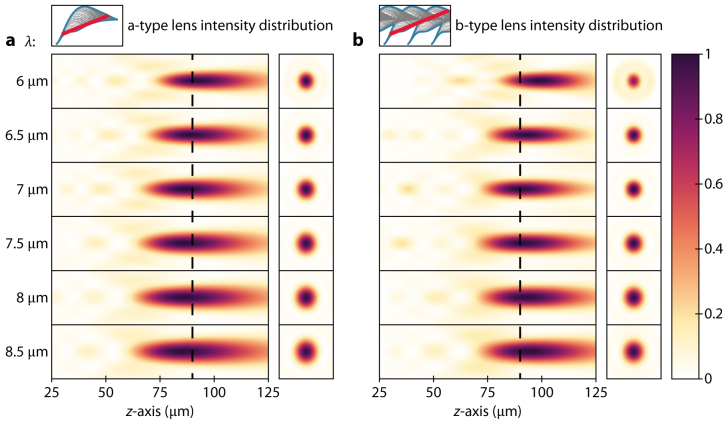
Intensity distributions and focal length shifts of metalenses in the wavelength range *λ* = 6–8.5 μm. (a, b) Simulated normalized intensity distributions of the designed a- and b-type metalens, respectively, and their associated point spread functions at the target focal length of *f* = 90 μm.

To verify the achromatic nature of the designed metalenses, the focal lengths at different wavelengths are determined by analyzing the intensity profiles (point spread functions) along the propagation direction (*z*-axis) of a continuous wave incident light beam (Figure 3). The a-type lens intensity distribution reveals chromatic aberrations are significantly reduced, over the entire bandwidth, with an observable focal length variation of no consequence (Figure 3(a)). This negligible focal length variation is further exemplified by investigating the point spread function at the target of *f* = 90 μm, where no defocusing is apparent. In fact, the outstanding suppression of chromatic aberrations suggests that the focal spot would maintain its profile for an appreciable increase of the characterization spectrum. Moreover, it is common to observe significant parasitic focal spots – partly due to an arbitrary design framework – meaning that the metalens is inaccurately focusing the incident light beam [26, 33, 36, 37]; however, such deficiencies are not evident using our methodology. Figure 3(b) complements these observations by displaying the intensity distribution for the b-type metalens, where a slight focal length variation but no parasitic focal spots are observed over the entire bandwidth. A smaller focal spot is observed in this case, attributable to the higher NA of the particular lens.

**Figure 4:**
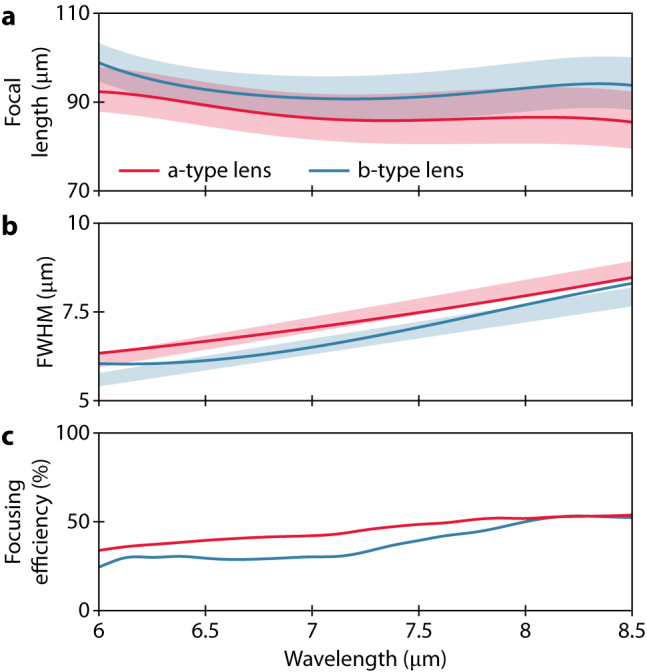
Figures of merit of the metalenses in the wavelength range *λ* = 6–8.5 μm. (a) Focal length as a function of wavelength. Shadings indicate the depth of field (DoF) – here defined as the region in which the intensity is higher than 95% of the value at the focal point. (b) Full width at half maximum (FWHM) of the point spread function at the focal spots, where shadings indicate the diffraction limit ranges that correspond to the observed focal length variations in (a). (c) Focusing efficiencies.

The exact focal length, at any given wavelength, corresponds to the z-coordinate possessing the highest intensity value. Figure 4(a) shows the simulated focal lengths of the metalenses and reveals that the foci are not perfectly established at the design focal point with a maximum deviation from the target approaching 10%. However, the variation of focal length, over the entire bandwidth, is insignificant considering that the designed focal point generally lies within the range of the depth of field (DoF), which is defined as the region in which the electric field intensity is higher than 95% of the value at the focal point.

Figure 4 summarizes other important figures of merit as well, including the focal spot size and the focusing efficiency. Both lenses display nearly ideal full width at half maximum (FWHM) values close to the theoretical diffraction limit as shown in Figure 4(b). Due to the larger NA of the b-type lens, it slightly outperforms the a-type lens. The shaded areas indicate the ranges of the diffraction limit that correspond to the observed focal spot variations. The focusing efficiency here is defined as the ratio of optical power enclosed in a 3FWHM area of the focal spot to the optical power of the incident beam. The remarkable localization of field power, at the desired focal point, leads to a focusing efficiency of 41% averaged over the entire bandwidth for both lenses with a maximum efficiency above 54% (Figure 4(c)). The variation of the focusing efficiency can be attributed to the transmission amplitude variations among the meta-elements, which may stem from neglecting higher-order modes in our model. Furthermore, the nearest-neighbor effects – i.e., optical response perturbations caused by adjacent meta-elements – will play a role in the slight variations. These effects combined lead to non-negligible unanticipated reflection which limits the focusing efficiencies. Correcting the focusing efficiencies with respect to the metalens transmission yields a normalized focusing efficiency over 80 % and 65% in the whole spectrum for the a- and b-type metalenses, respectively (see Supplementary information section S6). This implies that it is possible to further improve the focusing efficiency by suppressing the reflection loss. To minimize reflection, it is critical to match the wave impedance of the metalens to that of free space, which requires an elaborate design of meta-elements employing full-wave simulations. Nevertheless, we note that, while the efficiencies of the presented metalenses are not fully on par with monochromatic designs [20], [21], [22], they are comparable and even outperform other recent dielectric achromatic metalens demonstrations [23], [24], [25], [26], [27], [28], [29], [30], [31], [32], [33], [34], [35], [36], [37].

Additionally, our design methodology allows for the generation of a broadband focused vortex-beam with polarization-independence. In order to do so, the metasurface should incorporate the phase profile of a metalens (Eq. (1)) and a q-plate together:

(3) $$\varphi \left(r,\theta ,\omega \right)=-\frac{\omega }{c}\left(\sqrt{r^{2}+f^{2}}\right)+l\theta +C\left(\omega \right)\text{,}$$

**Figure 5:**
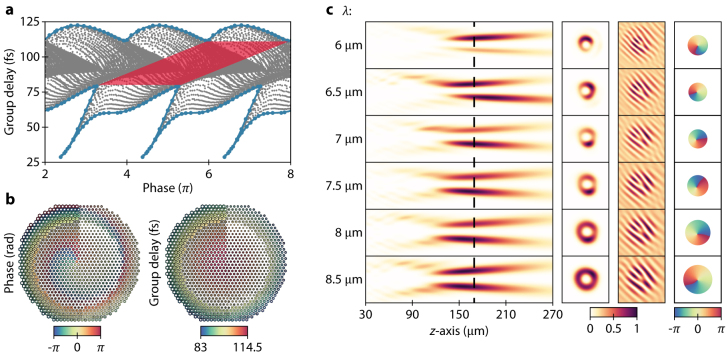
Framework for designing an achromatic metalens generating and focusing a vortex-beam. (a) Phase and group delay for all elements in the presented library distinctive by the grey dots, with the blue lines defining the upper and lower boundary of the library, and the red shaded area displaying the maximal phase range which the library can cover. (b) A metalens designed with the vortex-beam focusing framework, carrying OAM *l* = −1. The color overlays indicate the phase (left) and group delay (right) held by each element. (c) Simulated normalized intensity distributions of the designed metalens, its cross-section, interference pattern, and phase at the focal length of *f* = 165 μm.

Where *l* is the topological charge and θ=atan(y/x) is the azimuthal coordinate. In our framework, this equals a 2π broadening of the phase range line as illustrated in Figure 5(a). Figure 5(b) displays the phase and group delay profile of the designed focused vortex-beam generator with a diameter of *D* = 120 μm, focal length of *f* = 200 μm (NA = 0.29), and topological charge of *l* = −1.

The intensity distribution reveals that chromatic aberrations are significantly reduced over the entire bandwidth. However, a noteworthy difference between the desired and the achieved focal length is observed, as the focal point appears at *f* ≈ 165 μm (NA ≈ 0.34) while the vortex-beam still remains highly focused at the desired *f* = 200 μm (Figure 5C; Supplementary information section S7 shows the cross-sectional intensity profile of the vortex-beam at *f* = 200 μm). The origins of this shift are uncertain but may arise due to the phase profile imposed by Eq. (3), which prescribes a pronounced meta-element variation that could cause a considerable increase in nearest-neighbor effects, rendering the metalens defective. Furthermore, in this design we employ the “star” shaped meta-elements to fill in the library with low phase and high group delay elements, whose fundamental guided modes tend to possess more significant higher-order dispersion terms compared to other types of elements (Figure S1B). Moreover, the meta-elements with low phase and high group delay seem to support non-negligible excitations of higher-order modes as discussed in Supplementary information section S4. We speculate that these non-idealities – higher order modes and nonlinear dispersion terms –lead to small but noticeable sampling inaccuracies. At the focal point of *f* ≈ 165 μm the cross-sectional intensity profiles still reveal the characteristic doughnut-shape of vortex-beams, i.e., intensity singularity. To further manifest the helicity of the focused vortex-beam wavefront, we combine the electric field components with a linear phase ramp – which is similar to performing an interference experiment in practice – and fork dislocations are observed, verifying the phase singularity. Furthermore, the phase profile of the focal spot confirms this phase singularity. Our designed vortex-beam generating and focusing achromatic metalens rivals existing vortex generating metadevices in terms of bandwidth [16, 36, 37, 47], [48], [49].

## 3 Conclusions

In summary, we have demonstrated an analytical framework prescribing the exact phase range and appropriate spectral degree of freedom C(ω) of any achromatic metalens, based on the library used to design it. This framework, which can be implemented in all spectra, facilitates not only normal achromatic metalenses but also ones capable of focusing ultra-short optical pulses with no wavepacket distortion and generating achromatically focused vortex-beams. Capitalizing on our design scheme we present three devices – two types of achromatic lenses maximally utilizing their respective meta-element libraries and a lens generating and focusing a vortex-beam – exemplifying the efficacy of employing an analytical approach for metalens designing. The lenses exhibit exceptional correction of chromatic aberrations in the mid-infrared spectrum, while maintaining diffraction-limited focusing with an average focusing efficiency of 41% and a maximum efficiency over 54%. The framework presented in this paper clarifies the underlying physics involved in chromatic aberration correction and thus represents an important advance toward practical implementations of functional metalenses.

## 4 Methods

We performed three-dimensional FEM simulations using a commercial software package (COMSOL Multiphysics 5.4). Periodic boundary conditions are applied on the cross-sectional boundaries of the unit-cell, and the perfectly matched layer (PML) is used above and below the meta-element to truncate the simulation domain. To excite the dielectric meta-element, a plane wave impinges normally on the structure from the bottom.

We performed three-dimensional FDTD simulations using a commercial software package (Lumerical Inc.). The simulations inject a plane wave – in silicon – with normal incidence to the surface. A circular aperture (perfect electric conductor) was implemented between the source and metalens, limiting the area of injection to the exact metalens area. To truncate the air domains surrounding the metalens, PMLs were set as simulation boundaries – all boundaries were placed more than two wavelengths away from everything.

## Supporting Information

The following files are available free of charge. Supplementary information (Supplementary information.pdf).

## Abbreviations

MIR: mid-infrared
OAM: orbital angular momentum
FEM: finite-element method
NA: numerical aperture
FDTD: finite-difference time-domain
DoF: depth of field
FWHM: full width at half maximum

## Supplementary Material

Supplementary Material Details
