# Supplementary material for: Design framework for polarization-insensitive multifunctional achromatic metalenses
Source: Nanophotonics. 2022 Jan 4;11(3):583–91. doi: 10.1515/nanoph-2021-0638 (PMC11501271; doi:10.1515/nanoph-2021-0638)
Supplement: Supplementary file 1 — Supplementary Material Details [file j_nanoph-2021-0638_suppl.pdf]

Supplementary information
Design framework for polarization-insensitive multifunctional achromatic metalenses

Jacob T. Heiden[1] and Min Seok Jang[1,*]
[1]*School of Electrical Engineering, Korea Advanced Institute of Science and Technology (KAIST), Daejeon, Korea*

Contents

S1. State-of-art achromatic metalenses 1
S2. Meta-element library 2
S3. Metalens sampling 3
S4. Analysis of wavefront distortion 5
S5. Considerations when duplicating library 7
S6. Focusing efficiency correction 8
S7. Focused vortex-beam at design focal point 9
References 10

S1. State-of-art achromatic metalenses

Several performance metrics are important when designing an achromatic metalens, dependent on the intended application. A few things, however, are key: there is a strong desire for polarization-insensitivity, high focusing efficiency, and bandwidth. The characteristics in various state-of-art broadband achromatic metalenses are summarized in table S1.

Table S1. Summary of performance characteristics for achromatic metalenses. NA, numerical aperture. The abbreviations (e) and (s) specify whether the peak efficiency is measured in experiment or simulated, respectively. The polarization sensitivity catalogs what incident polarization can be focused.

Diameter (μm)	NA	Material	Bandwidth (μm)	Peak efficiency	Polarization	Ref.
56	0.27	$Au/SiO_2/Au$	1.2-1.65	12% (e)	Circular	S1
220	0.02	TiO_2	0.47-0.67	20% (e)	Circular	S2
50	0.106	GaN	0.4-0.66	67% (e)	Circular	S3
12.5	0.9	TiO_2	0.45-0.7	33% (s)	Linear	S4
12.5	0.1	TiO_2	0.45-0.7	80% (s)	Linear	S4
200	0.2	$TiO2/SiO_2/Al$	0.49-0.55	20% (e)	Insensitive	S5
26.4	0.2	TiO_2	0.4-0.7	36% (e)	Insensitive	S6
200	0.45	Si	3.5-4.75	45% (e)	Insensitive	S7
200	0.13	α-Si	1.2-1.65	55% (e)	Insensitive	S8
20	0.077	TiO_2	0.64-1.2	75% (e)	Insensitive	S9
30	0.24	TiO_2	0.65-1	85% (e)	Insensitive	S10
100	0.49	Si	6-8.5	54% (s)	Insensitive	This work
120	0.55	Si	6-8.5	54% (s)	Insensitive	This work

S2. Meta-element library

It is mentioned in the main paper that a waveguide model is employed to assemble the meta-element library. This choice is made to simplify and speed up the process of generating a library with thousands of meta-elements. The model considers each element as a truncated waveguide with a fundamental mode frequency-dependent effective refractive index $n_{\text{eff}}(\omega)$. Accordingly, the phase of the propagating light is approximated to:

$$\varphi(\omega) = \frac{\omega}{c} n_{\text{eff}}(\omega) h, \tag{S1}$$

where h is the height of the waveguide and c is the speed of light in vacuum. The derivative of Eq. (S1), with respect to angular frequency, yields the group delay:

$$\frac{\partial \varphi(\omega)}{\partial \omega} = \frac{1}{c} n_{\text{eff}}(\omega) h + \frac{\omega}{c} \frac{\partial n_{\text{eff}}(\omega)}{\partial \omega} h. \tag{S2}$$

Analyzing a set of the meta-elements used to create the metalenses presented in the main paper reveals how the phase and group delay library materializes with the waveguide model. Figure S1a indicates the respective meta-element positions in the library and Fig. S1b depicts the effective refractive index of the fundamental mode, of the selected meta-elements, as a function of wavelength – all the elements analyzed are used in the presented metalenses.

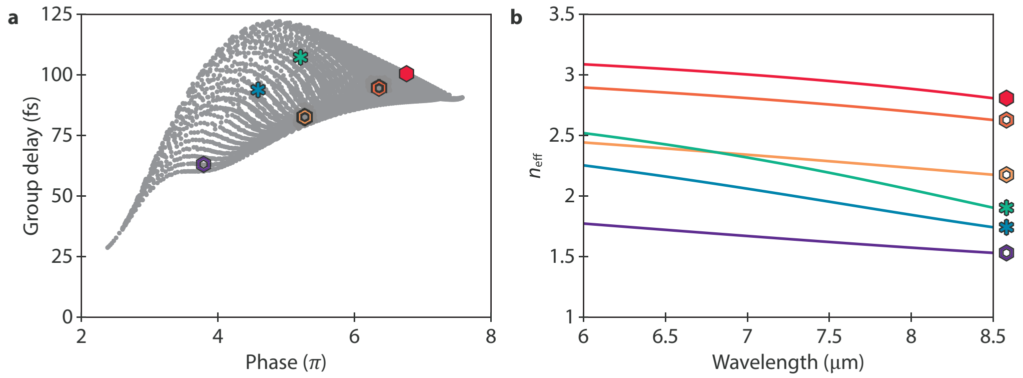

Figure S1. Effective refractive indices meta-elements. **a**. Phase and group delay for all elements in our library over the $\lambda = 6 - 8.5$ μm range. The colored element logos display the element type and library position of the elements analyzed. **b**. Effective refractive index n_{eff} as a function of wavelength of the elements displayed in the library.

By definition, a low n_{eff} and $\partial n_{\text{eff}}/\partial\omega$ combined yields a low phase and group delay, while a high n_{eff} and $\partial n_{\text{eff}}/\partial\omega$ combined yields a high phase and group delay. However, in order to achieve a low phase and high group delay, a low n_{eff} but significantly higher $\partial n_{\text{eff}}/\partial\omega$ – than needed for high phase and group delay – is required. Employing only the fundamental mode is, generally, a sufficiently accurate method for obtaining the phase of a meta-element [S2; S8]; which is exemplified by full-wave simulations in the main paper. The fundamental mode is largely invariable, meaning that by this model $\varphi(\omega)$ can be linearly interpolated

with an average root-mean-square error of 1 % and the largest deviation from this linear interpolation is an error of 4.7 %. Effectively, this renders the group delay dispersion zero, simplifying the design framework. Examining a selection of meta-elements used in the actual metalenses verifies this claim (same elements as examined in Fig. 1d, Fig. S2).

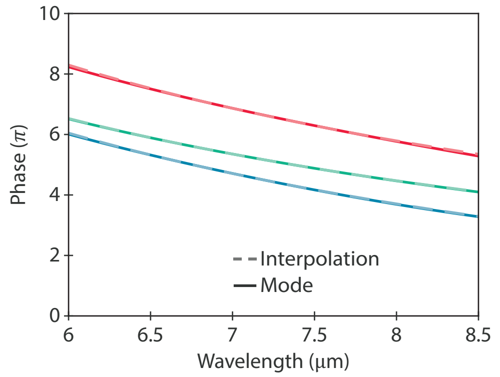

Figure S2. Linear interpolation of phase. A comparison of the phase response for different meta-elements from eigenmode solutions versus a linear interpolation. The red curve represents a 'solid' element; the green a 'hole' element; and the blue a 'star' element.

S3. Metalens sampling

As described in the main paper, the upper and lower boundaries of the library can be described as polynomials, and since the relation between the required phase and group delay is linear (monotonically increasing) a sampling line, enclosed in the library, can be made. We here show Fig. 2a again for reference (Fig. S3).

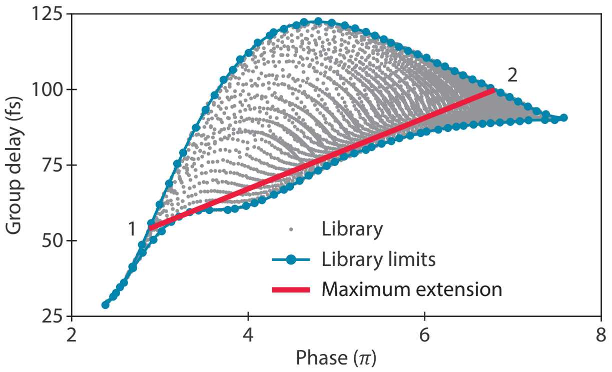

Figure S3. Design framework. Phase and group delay for all elements in the presented library, distinctive by the color grey, the blue lines define the upper and lower boundary of the library, and the red line displays the maximal phase range which the library can cover.

The library generated with the waveguide model contains 4040 elements; however, it is clearly still sparse, especially in the low phase parts of the library. Therefore, when sampling metalenses we incorporate an interpolation between elements. More specifically, we find the

meta-element possessing properties most like the intended and create a grid of other meta-elements surrounding it in the phase vs. group delay diagram, then we linearly interpolate between these, effectively rendering our library infinitely fine. Following this approach, each element used in the metalenses lies exactly on the red design line.

Utilizing the presented framework, the library of meta-elements is fitted as described above. The metalenses presented in the main paper include an a-type lens with $f = 90$ μm and $D = 100$ μm (Fig. S4a), a b-type lens with $f = 90$ μm and $D = 120$ μm (Fig. S4b), and a vortex-generating lens with $f = 200$ μm and $D = 120$ μm (Fig. S4c). The fitting clearly demonstrates the impact of selecting the correct $C(\omega)$ values, phase range, and sampling scheme.

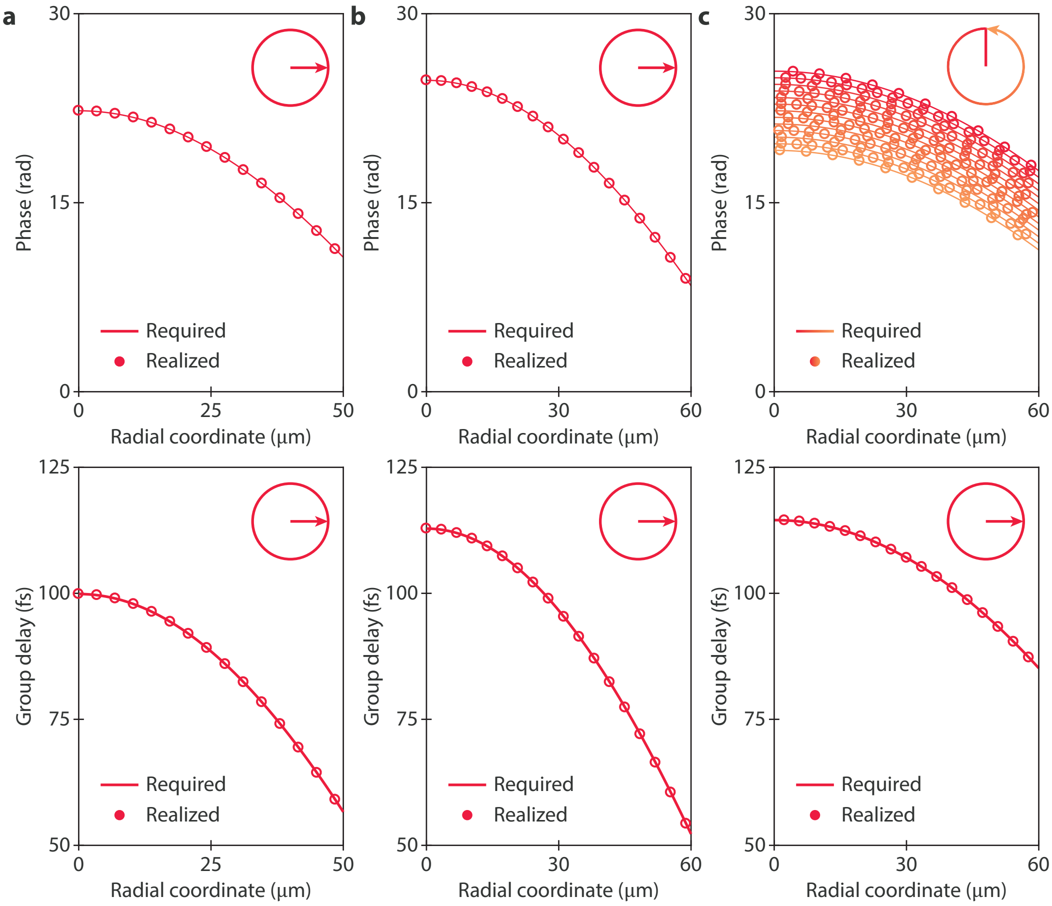

Figure S4. Phase sampling of the metalenses. The line(s) indicates the required phase and group delay at each radial coordinate for achromatic focusing and the circles indicate the realized values. **a**. a-type lens with $f = 90$ μm and $D = 100$ μm, **b**. b-type beam lens with $f = 90$ μm and $D = 120$ μm. **c**. a vortex-generating lens with $f = 200$ μm and $D = 120$ μm. The insets of a circle and arrow indicate the sampling line.

S4. Analysis of wavefront distortion

As demonstrated in the main paper (Fig. 2), the b-type metalens, which is based on the extended library, shows slightly higher wavefront distortion compared to the a-type lens based on the non-extended library. To see how the 2π phase jump is manifested in the wave propagation, we first compare the electric field (x-component) snapshots of the two metalenses for a 25 fs optical pulse input as shown in figure S5. At the position of the phase jump, which is indicated by the black arrows in figure S5b, the wavefront of the b-type lens shows some irregularities and discontinuities that would partially cause the distortion at the focal point. In addition, the region between the black arrows, which is occupied by the meta-elements from the extended library, shows premature transmission of waves even though the elements in this region are supposed to exhibit longer group delays than those in the peripheral region. We speculate that the early arriving wave components shown in figure 2f originate from this non-ideal premature transmission of waves.

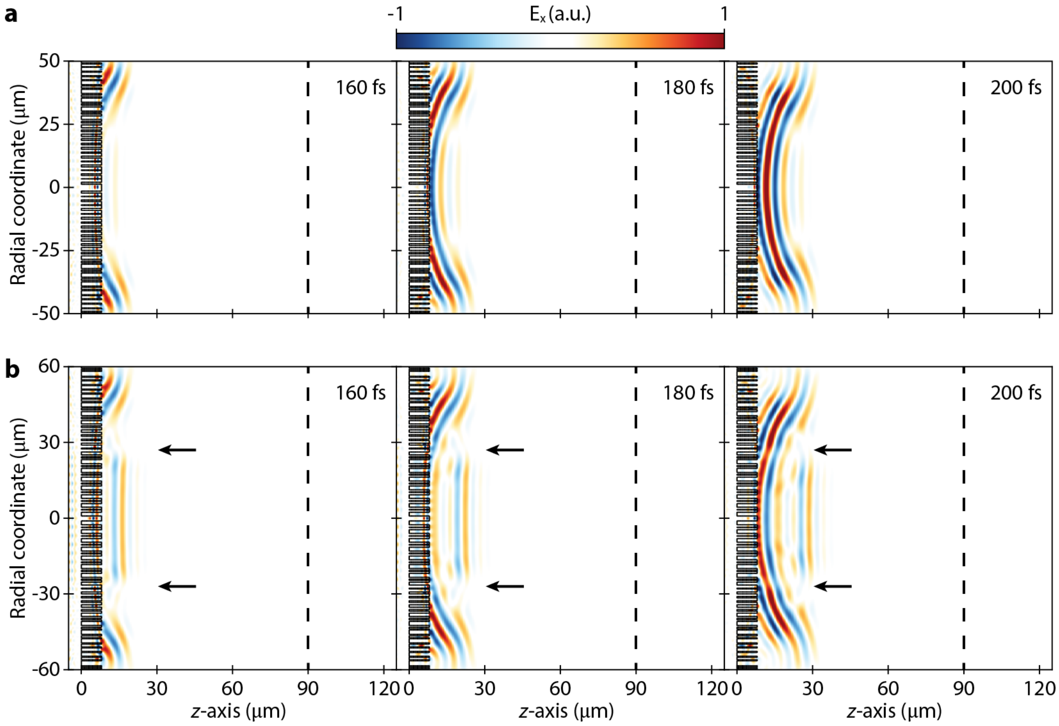

Figure S5. Generation of spherical wavefront. a,b. Simulated electric fields (x-component) of a 25 fs optical pulse under the formation of a spherical wavefront when transmitting through the a- or b-type metalens, respectively. The arrows point to the abrupt phase shift.

To further investigate the issue of premature transmission, we performed additional full-wave simulations to check the actual wave transmission through each meta-element used in the b-type lens. Here we pick an individual meta-element in the library, form a metasurface by creating an infinite array of the meta-element, and simulate how a wave packet propagates through the metasurface. Figure S6 shows the resulting electric field as a function of time

recorded at the 90 µm distance from the meta-element array. The elements used in the a-type lens all display a well-preserved wavepacket (Fig. S6b,d,e). In contrast, the elements from the extended part (Fig. S6c,f), produce significantly elongated wave packets with distortion, possibly due to the excitation of higher-order modes and non-linear dispersion of the fundamental mode. As displayed in figure S6g-i the same tendency is observed for "star" shaped elements used for the focused vortex-beam generator.

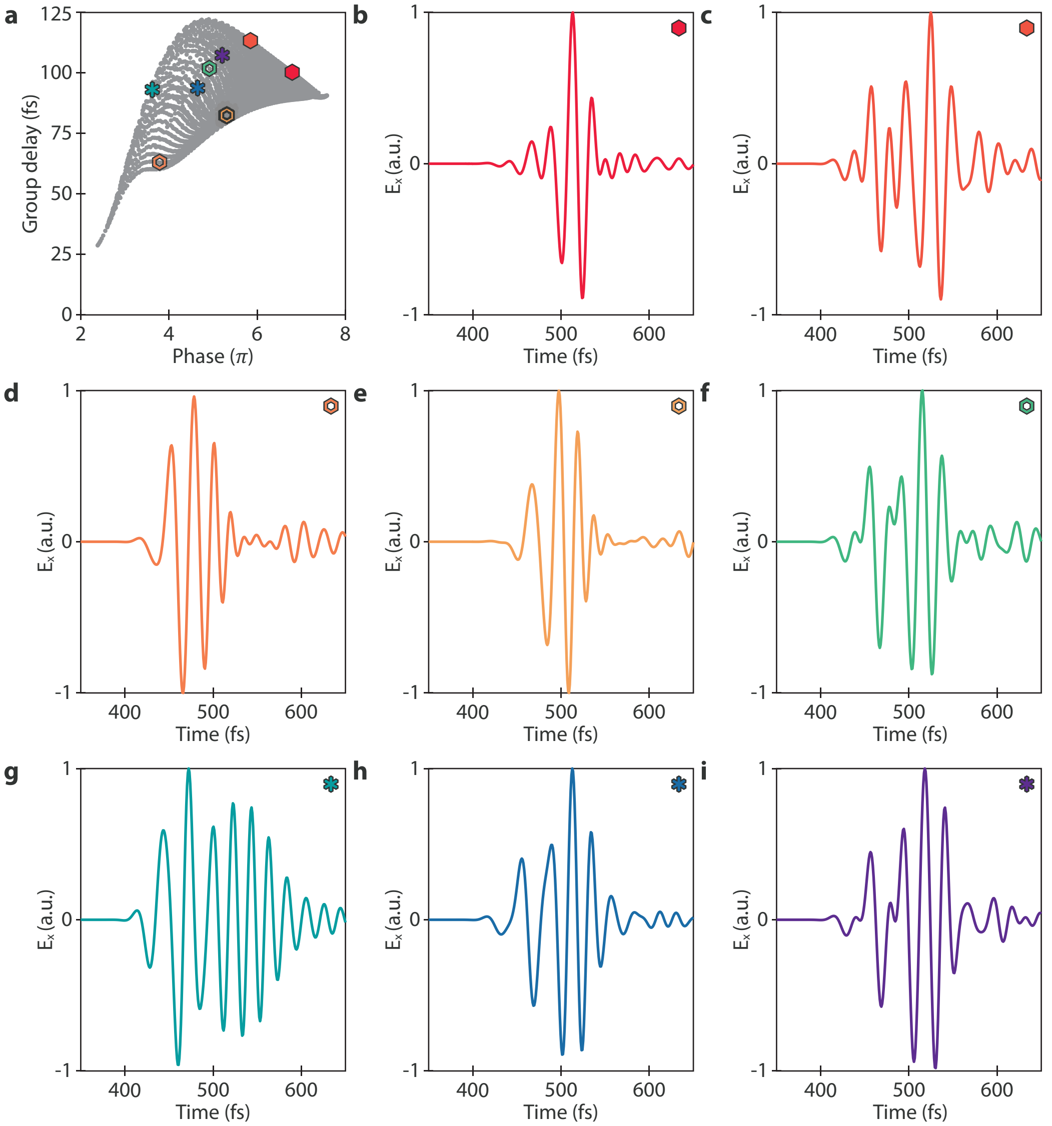

Figure S6. Meta-element wavepacket arrival time and distortion. **a**. Phase and group delay for all elements in our library over the $\lambda = 6 - 8.5$ µm range. The colored element logos display the element type and library position of the elements analyzed. **b**-**i**. Simulated normalized electric fields (x-component) at the focal point distance as a function of time, for individual meta-elements.

S5. Considerations when duplicating library

In the main paper, we make the rational choice to only employ the duplicated library when the original is out of reach. Here, we demonstrate the ramifications of following a different design strategy. Figure S7a displays, with a green line, the phase range which the duplicated library can cover; we design a new metalens with the same specification as the b-type lens system – i.e., $f = 90$ µm and $D = 120$ µm (NA $= 0.55$). Similarly, the time-dependent propagation of a 25 fs optical pulse impinging on the metalens is simulated using FDTD. Figure S7b shows the electric field snapshot of the lens systems taken at $t = 485$ fs. The aberration, taking the form of wavefront elongation at the focal point, is significantly enhanced. This is further exemplified in Fig. S7c showing the electric field at the focal point in time. The field of the metalens is split into the contributions from the red and green line parts of the lens; the peculiar wavefront aberration observed for the b-type lens, in the main paper, is amplified as a cause of the issues of the higher order modes and dispersion terms discussed in section S4. Therefore, considering other library duplication strategies over the one presented in the main paper is inadvisable.

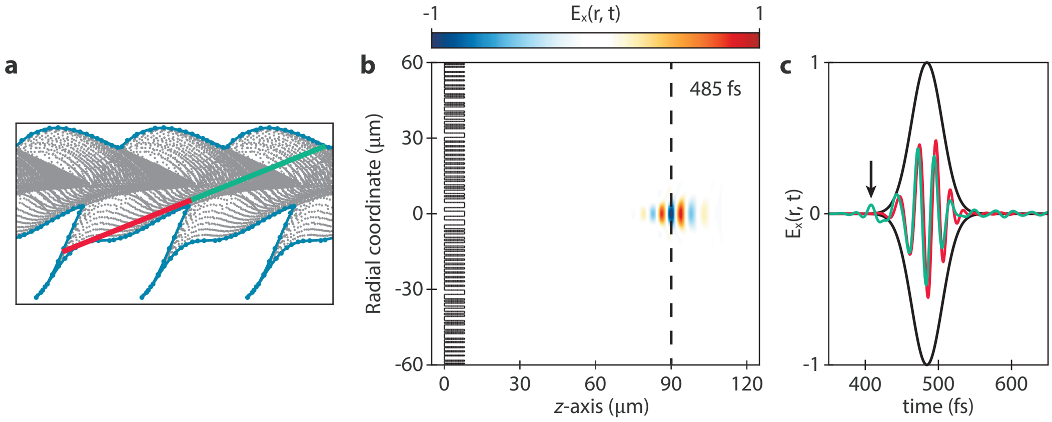

Figure S7. Considerations when duplicating library. **a**. Phase and group delay for all elements in the presented duplicated library, distinctive by the color grey, where the blue lines define the upper and lower boundaries of the library, and the red line displays the maximal phase range which the library can cover. **b**. Simulated normalized electric field (x-component) of a 25 fs optical pulse as it is focused at the focal point ($f = 90$ µm). **c**. Simulated normalized electric field (x-component) at the focal point as a function of time; the pulse is split into the component attributions of the red and green line from (a).

S6. Focusing efficiency correction

Since our metalens design framework takes no account of transmission in the individual meta-elements and neglects higher-order modes, the focusing efficiency of the metalenses is not optimized and is highly frequency dependent – as demonstrated in the main paper (Fig. 4c). However, normalizing the focused power to the transmitted power, of the respective metalens, yields a higher and more stable focusing efficiency of more than 80 % and 65 % for the a- and b-type metalenses, respectively (Fig. S8).

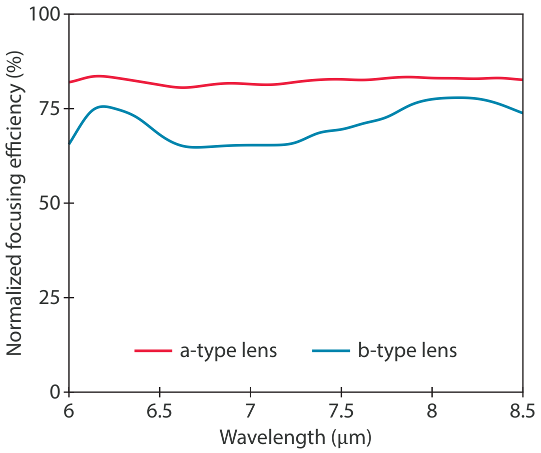

Figure S8. Focusing efficiency correction. The focusing efficiency of the a- and b-type metalenses, from the main paper, normalized to their respective transmissions.

S7. Focused vortex-beam at design focal point

The achromatic vortex-beam generating metalens, presented in the main paper, displays a notable difference in the design focal length to the achieved, as the focal point appears at $f \approx 165$ µm (NA ≈ 0.34). However, as displayed in Fig. S9 the vortex-beam still remains highly focused at the desired $f = 200$ µm. The classic fork dislocations – which stems from interference between the electric field components and a linear phase ramp – are still observable, and the phase profile at $f = 200$ µm still clearly demonstrates the phase singularity.

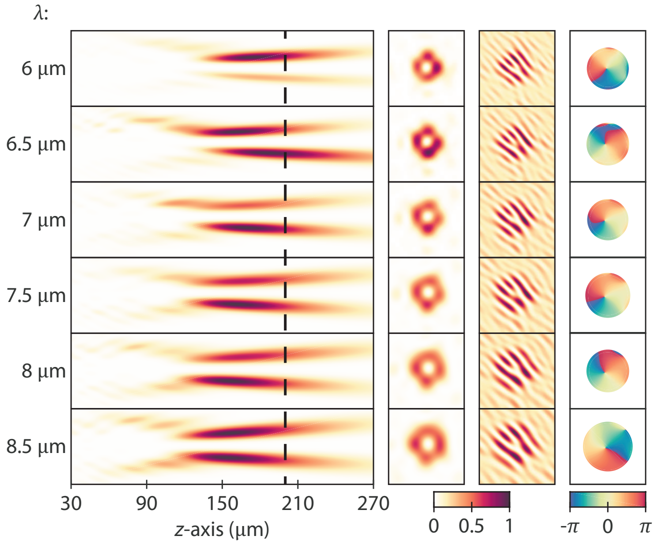

Figure S9. Vortex-beam focusing at design focal point. Simulated normalized intensity distributions of the designed metalens, its point spread function, interference pattern, and phase at the focal length of $f = 200$ µm.

References

* jang.minseok@kaist.ac.kr

[S1] S. Wang, P. C. Wu, V.-C. Su, Y.-C. Lai, C. H. Chu, J.-W. Chen, S.-H. Lu, J. Chen, B. Xu, C.-H. Kuan, T. Li, S. Zhu, and D. P. Tsai, *Nat. Commun.* **8**, 187 (2017).

[S2] W. T. Chen, A. Y. Zhu, V. Sanjeev, M. Khorasaninejad, Z. Shi, E. Lee, and F. Capasso, *Nat. Nanotechnol.* **13**, 220 (2018).

[S3] S. Wang, P. C. Wu, V.-C. Su, Y.-C. Lai, M.-K. Chen, H. Y. Kuo, B. H. Chen, Y. H. Chen, T.-T. Huang, J.-H. Wang, R.-M. Lin, C.-H. Kuan, T. Li, Z. Wang, S. Zhu, and D. P. Tsai, *Nat. Nanotechnol.* **13**, 227 (2018).

[S4] H. Chung and O. D. Miller, *Opt. Express* **28**, 6945 (2020).

[S5] M. Khorasaninejad, Z. Shi, A. Y. Zhu, W. T. Chen, V. Sanjeev, A. Zaidi, and F. Capasso, *Nano Lett.* **17**, 1819 (2017).

[S6] W. T. Chen, A. Y. Zhu, J. Sisler, Z. Bharwani, and F. Capasso, *Nat. Commun.* **10**, 355 (2019).

[S7] K. Ou, F. Yu, G. Li, W. Wang, J. Chen, A. E. Miroshnichenko, L. Huang, T. Li, Z. Li, X. Chen, and W. Lu, *Laser Photonics Rev.* **15**, 2100020 (2021).

[S8] S. Shrestha, A. C. Overvig, M. Lu, A. Stein, and N. Yu, *Light Sci. Appl.* **7**, 85 (2018).

[S9] A. Ndao, L. Hsu, J. Ha, J.-H. Park, C. Chang-Hasnain, and Boubacar Kanté, *Nat. Commun.* **11**, 3205 (2020).

[S10] Y. Wang, Q. Chen, W. Yang, Z. Ji, L. Jin, X. Ma, Q. Song, A. Boltasseva, J. Han, V. M. Shalaev, and S. Xiao, *Nat. Commun.* **12**, 5560 (2021).
